# Establishing a General Atomistic Model for the Stratum Corneum Lipid Matrix Based on Experimental Data for Skin Permeation Studies

**DOI:** 10.3390/ijms26020674

**Published:** 2025-01-15

**Authors:** Navaneethan Radhakrishnan, Sunil C. Kaul, Renu Wadhwa, Lee-Wei Yang, Durai Sundar

**Affiliations:** 1 Department of Biochemical Engineering and Biotechnology, Indian Institute of Technology (IIT) Delhi, New Delhi 110016, India; navaneethan@iitdalumni.com; 2 Institute of Bioinformatics and Structural Biology, National Tsing Hua University, Hsinchu 300044, Taiwan; lwyang@life.nthu.edu.tw; 3 AIST-INDIA DAILAB, National Institute of Advanced Industrial Science & Technology (AIST), Tsukuba 305 8565, Japan; s-kaul@aist.go.jp (S.C.K.); renu-wadhwa@aist.go.jp (R.W.); 4 Bioinformatics Program, Institute of Information Sciences, Academia Sinica, Taipei 115201, Taiwan; 5 Physics Division, National Center for Theoretical Sciences, Taipei 106319, Taiwan; 6 Biomedical Artificial Intelligence PhD Program, National Tsing Hua University, Hsinchu 300044, Taiwan; 7 Yardi School of Artificial Intelligence, Indian Institute of Technology (IIT) Delhi, New Delhi 110016, India; 8 Institute of Bioinformatics and Applied Biotechnology (IBAB), Bengaluru 560100, India

**Keywords:** skin permeability, stratum corneum lipid matrix, molecular dynamics (MD) simulation, potential of mean force, permeability coefficient, permeation enhancers

## Abstract

Understanding the permeation of drugs through the intercellular lipid matrix of the stratum corneum layer of skin is crucial for effective transdermal delivery. Molecular dynamics simulations can provide molecular insights into the permeation process. In this study, we developed a new atomistic model representing the multilamellar arrangement of lipids in the stratum corneum intercellular space for permeation studies. The model was built using ceramides in extended conformation as the backbone along with free fatty acids and cholesterol. The properties of the equilibrated model were in agreement with the neutron scattering data and hydration behavior previously reported in the literature. The permeability of molecules, such as water, benzene and estradiol, and the molecular mechanism of action of permeation enhancers, such as eucalyptol and limonene, were evaluated using the model. The new model can be reliably used for studying the permeation of small molecules and for gaining mechanistic insights into the action of permeation enhancers.

## 1. Introduction

Different chemical formulations have traditionally been applied onto skin for pharmaceutical and cosmetic purposes to induce systemic or local effects. Transdermal delivery refers to the non-invasive and painless method in which drugs can be delivered by applying the formulation onto intact skin. In clinical settings, transdermal delivery systems are advantageous over oral drug delivery in that drugs delivered through transdermal routes skip first-pass metabolism via liver and the adverse gastrointestinal environment and hence can have increased plasma half-life times [1]. Also, a more controlled delivery is possible in transdermal administration. Improved bioavailability as well as patient compliance make transdermal drug delivery an attractive choice for delivery of small molecule drugs in diseases like cancer [1].

Drugs applied to the skin have to cross the permeability barrier of the skin to reach the target tissues. The stratum corneum—the outermost layer of the epidermis—forms the skin’s permeability barrier, protecting the underlying tissue against infection from physical, chemical and biological factors in the environment [2]. It is ~10–15 cells deep (~10–20 µm) and forms the primary barrier for entry of drug molecules [3]. The barrier properties of the stratum corneum are attributed to the rigid arrangement of keratin-filled cells called corneocytes embedded in an intercellular lipid matrix [2]. The traversal of drugs through the stratum corneum can occur through passive or active mechanisms [4]. Passive permeation denotes the movement of a substance across the stratum corneum without the aid of an external force or energy input, whereas active permeation involves the use of external forces or energy to enhance the transport through the skin [4]. In passive permeation, the intercellular lipid matrix offers a continuous path through the stratum corneum [5]. This results in passive transport of the drug through the intercellular lipid matrix, the key determinant of permeability of any drug [5]. Hence, studying the transport of drugs across the lipid matrix of the stratum corneum is crucial for transdermal drug delivery.

The lipid matrix of the stratum corneum mainly consists of ceramides, free fatty acids and cholesterols in a ratio of 1:1:1 [6]. Ceramides are the two-tailed lipids of the stratum corneum, with a sphingosine tail and a fatty acid tail. There are several kinds of ceramides and free fatty acids in the stratum corneum. Previous reports have given a detailed account of the proportion of different types of lipid molecules and their length distribution [7,8,9]. The composition of the stratum corneum lipid matrix has been well studied [7,8,9], but the spatial organization of stratum corneum lipids has not been elucidated in a comprehensive manner. Several models have been proposed for the organization of lipids in the stratum corneum intercellular space based on data from X-ray scattering or electron microscopic experiments [10,11,12,13,14,15,16,17]. According to these studies, the intercellular lipids of the stratum corneum are stacked in a multilamellar arrangement with alternating hydrocarbon and polar regions [10,11,12,13,14,15,16].

The passive transport of a drug through the stratum corneum lipid matrix is dependent on the physicochemical properties of the drug, such as lipophilicity, aqueous solubility and molecular size [18]. To extend the range of molecules that can be transdermally delivered with good fluxes, different active and passive permeation enhancement strategies have been proposed [19]. Among them, the use of chemical permeation enhancers is a widely studied strategy [20]. Chemical permeation enhancers are molecules that penetrate the skin and reversibly compromise the barrier function of the stratum corneum, allowing or enhancing the permeation of active compounds [18,21]. The ways in which different permeation enhancers alter the structure of the stratum corneum lipid matrix vary, and the potency of a permeation enhancer tends to be dependent on the nature of the drug molecule as well [22]. Hence, it is not straightforward to make a rational choice of a permeation enhancer for any drug. Understanding the molecular mechanism of action of a permeation enhancer aids in finding suitable accompanying classes of drugs. Although many compounds have been explored as permeation enhancers, the molecular details of action have been studied for only a few of them [21,22].

In vitro and in vivo methods for assessing the skin permeability of molecules are cost-intensive and raise ethical and regulatory concerns. Empirical methods like quantitative structure property relationship (QSPR) models have been shown to predict permeability with good accuracy [23]. However, these mathematical models cannot be used in the context of studying complex molecular mechanisms involved in drug permeation [24]. In silico molecular models provide a promising way to study the molecular mechanisms involved in the permeation of drugs through complex lipid systems. Molecular dynamics (MD) simulations have been used to predict the permeability of drugs through cell membranes using simple lipid bilayer models and through stratum corneum lipid systems as well [25,26,27,28]. In these studies, the stratum corneum lipid matrix has often been modeled as a lipid bilayer immersed in bulk water, with the polar groups of the free fatty acids and the cholesterol molecules contacting the polar groups of ceramides that are in hairpin conformation (Supporting Information: Figure S1A) [27,29,30,31]. While the interlayer spacing of bilayers composed of lipids in hairpin conformation will be highly sensitive to water content [32], recent studies have highlighted that the interlayer spacing and overall structure of the stratum corneum lipid matrix are unaffected by hydration [33,34,35,36,37]. This non-swelling behavior has been attributed to the presence of the ceramides in extended conformation (Supporting Information: Figure S1B) [15,32]. When two-tailed lipids like ceramides are present in hairpin conformation, water can penetrate the space between the polar head groups of lipid lamellae, causing the lamellae to swell (Supporting Information: Figure S1C) [32]. In the case of the extended conformation, this swelling will not be possible (Supporting Information: Figure S1D) [32]. The presence of the extended conformation of ceramides in the stratum corneum has been suggested by different studies [12,15,33,34,38,39]. In this study, we used extended ceramides (Supporting Information: Figure S1B) as the backbone to model the stratum corneum lipids in a multilamellar arrangement. The built model was validated with data from previous neutron diffraction studies [36,38], and its utility for permeation studies was demonstrated.

**Figure 1:**
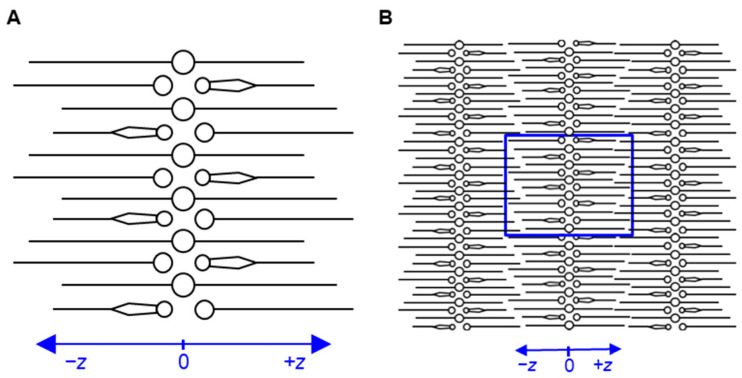
(**A**) Schematic representation of the arrangement of lipids in the built model of the stratum corneum lipid matrix. (**B**) Schematic representation with periodic images of the simulation box. Circles denote the polar groups of lipids; lines denote the hydrophobic tails; two-tailed representations denote ceramides; one-tailed representations denote free fatty acids; and one-tailed necktie-shaped representation denotes cholesterol. In the representation of ceramides, the shorter line denotes the sphingosine tail, and the longer line denotes the fatty acid tail. Water molecules are not shown.

The model was built using ceramides, free fatty acids and cholesterol in a ratio of 1:1:1. The schematic representation of the arrangement of lipids in the built model is shown in Figure 1. The ceramide head groups are in the center of the simulation box along the *Z*-axis, with the hydrocarbon tails pointing toward the periphery. The polar groups of free fatty acids and cholesterol are in contact with the polar groups of ceramides. The water molecules are packed near the polar groups of the lipid molecules. The system was periodic in all directions. With the absence of bulk water in the simulation box, the model can be considered to be in a multilamellar arrangement by virtue of the periodic boundary conditions. MD simulations were performed to equilibrate and study the properties of the model. The prepared model was used to compute the permeability of compounds like water, benzene and estradiol. The results were compared with previous MD studies on skin permeability [29,40]. Furthermore, the mechanism of action of permeation enhancers like eucalyptol and limonene was studied using the model.

## 2. Results

### 2.1. Developed Molecular Model of Stratum Corneum Lipid Matrix

**Figure 2:**
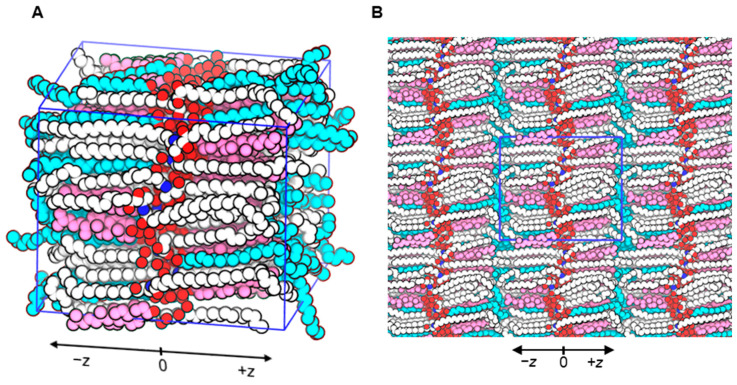
(**A**) Snapshot of the built model during production simulation. (**B**) Snapshot of the built model during production simulation with periodic images of the simulation box. Oxygen atoms are shown in red, nitrogen atoms in blue, carbon atoms of ceramides in white, carbon atoms of lignoceric acid in cyan, carbon atoms of cholesterol in magenta, and hydrogen atoms are not shown.

**Figure 3:**
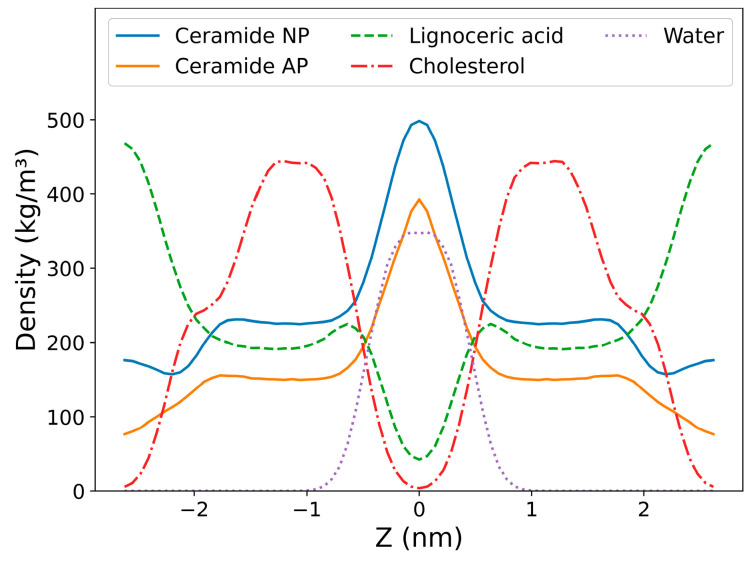
Densities of all lipid molecules and water molecules in the system along the direction of the bilayer normal. On the horizontal axis, ‘0’ indicates the center of the polar groups.

A new molecular model representing the arrangement of lipids in the stratum corneum intercellular space was built. Only the predominant and well-studied ceramides—ceramide NP (phytosphingosine) and ceramide AP (α-hydoxy phytosphingosine) at a ratio of 3:2—and the common free fatty acid in the stratum corneum (lignoceric acid) were used in the model to approximately mimic the experimental reports [7,8,9]. Similarly, the length of the fatty acid chains of the ceramides was 24 carbons, and they were saturated. Based on the estimation reported in neutron diffraction studies [38], 1.9 water/lipid was used. Figure 2 shows a snapshot of the model during production simulation. The ceramide head groups lie in the center of the simulation box, as indicated by the peaks in the density curves of ceramides along the *Z* direction (Figure 3). The head groups of ceramides form a single peak, whereas the polar groups of lignoceric acid and cholesterol form two distinct peaks.

**Figure 4:**
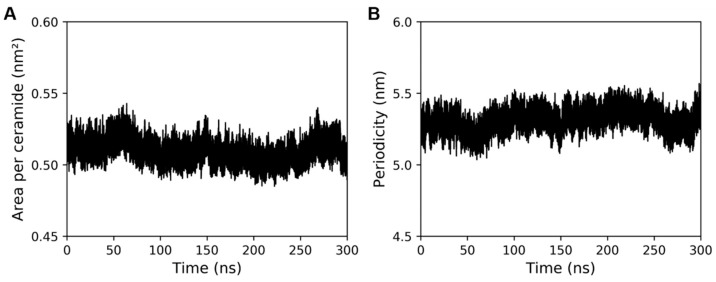
Properties of the built model of the stratum corneum lipid matrix during production simulation. (**A**) Area per ceramide, (**B**) Periodicity.

The convergence of system properties like periodicity and average cross-sectional area per ceramide was assessed to check whether the system had attained equilibrium. Periodicity refers to the distance between the lamellar phases that are formed by the intercellular lipids of the stratum corneum. In our system, this periodicity is the length of the simulation box in the *Z* direction. Figure 4 shows the time-dependent values of these properties during the production run. As there were no noticeable drifts in these values, the system was considered to be in a converged state. Furthermore, to check whether the ceramides with extended conformation were stable, the angles between the fatty acid tails and sphingosine tails of ceramides, ‘*θ*’, were calculated during production simulation.

**Figure 5:**
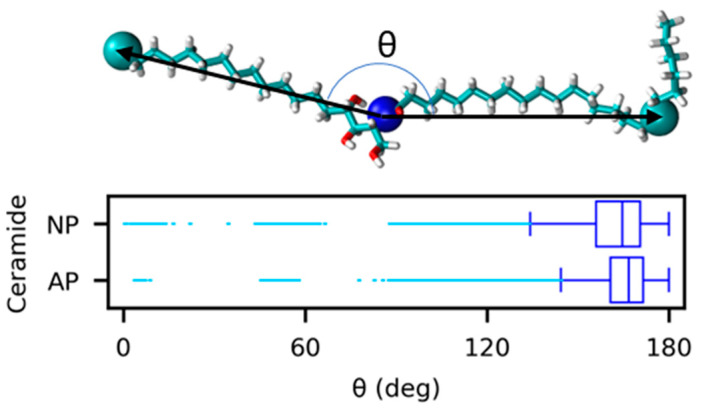
Angle distributions between the two tails of ceramide NP and ceramide AP molecules. *θ* indicates the angle between the sphingosine tails and fatty acid tails of ceramide molecules. The ‘*θ*’ angles between the sphingosine tails and fatty acid tails of the ceramides were calculated from the positions of the nitrogen atom and C18 atoms in the sphingosine tails and fatty acid tails of each ceramide molecule.

The box plot in Figure 5 shows the distribution of the calculated *θ* for ceramide NP and ceramide AP during production. The range of *θ*, excluding outliers, is >120° for both ceramide types, indicating that extended conformation prevailed throughout the production simulation.

To ensure that the equilibration was sufficient, two more replicas were studied from different initial configurations. The replicas were equilibrated using the same protocol, and the production simulation was performed for 300 ns. There were no noticeable drifts in area per ceramide and periodicity during the production run (Supporting Information: Figure S2). The average periodicity and the average cross-sectional area per ceramide during the production run were 5.3 nm and 0.51 nm^2^, respectively, for all three systems. The range of *θ*, excluding outliers, was >120° for both ceramide types in the replicas (Supporting Information: Figure S3). The properties of the three systems equilibrated from different initial configurations were consistent, indicating that the studied properties were not dependent on the initial configuration. To test whether system properties were influenced by the small size (120 lipids) of the system, another larger system with 360 lipids was built and equilibrated, and properties like area per ceramide and periodicity were studied. The average periodicity and the average cross-sectional area per ceramide during the 100 ns production run were 5.3 nm and 0.51 nm^2^, respectively (Supporting Information: Figure S4). The values were the same as the values calculated for the 120 lipid systems, indicating that the smaller size of the system did not influence the studied system properties.

### 2.2. Comparison of Model Properties with Experimental Observations from the Literature

**Figure 6:**
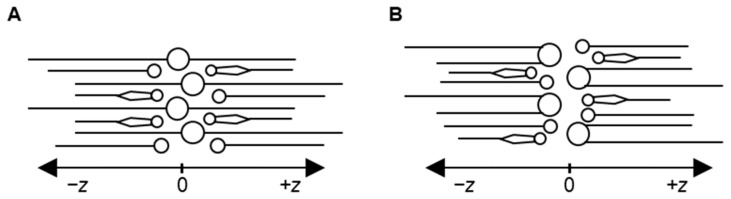
Schematic representation showing the organization of ceramides in (**A**) the extended-ceramide system and (**B**) the hairpin-ceramide system. Circles denote the polar groups of lipids; lines denote the hydrophobic tails; two-tailed representations denote ceramides; one-tailed representations denote free fatty acids; and one-tailed necktie-shaped representation denotes cholesterol. In the representation of ceramides, the shorter line denotes the sphingosine tail, and the longer line denotes the fatty acid tail. Water molecules are not shown.

**Figure 7:**
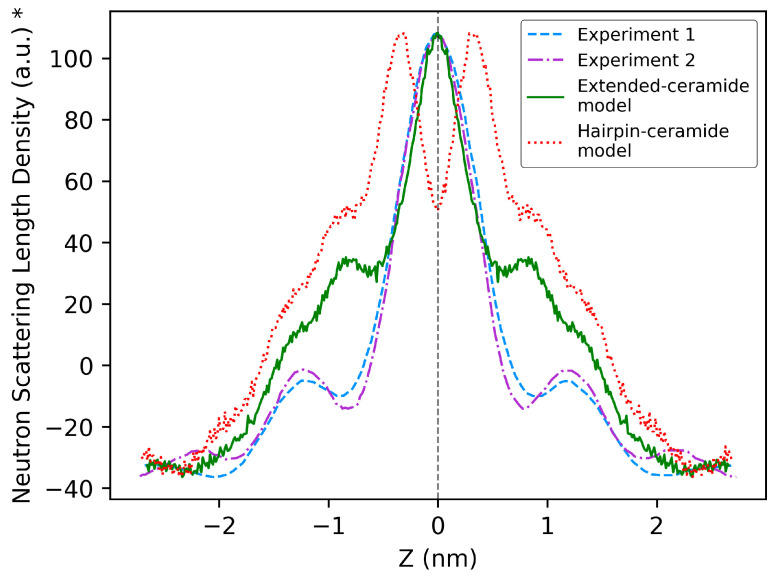
Comparison of neutron scattering length density profiles with experiments. Experiment 1: Groen et al., 2011 [38]; Experiment 2: Schmitt et al., 2018 [36]. On the horizontal axis, the value of 0 indicates the center of the polar groups. The profile of one periodic cell was plotted. * The scattering length density values were normalized. The profiles were recentered to position the center of the polar region at *z* = 0.

The average value of periodicity or lamellar repeat distance of the built model measured during the production run was 5.31 nm. This value lies in the range of 4–6 nm, as observed in experiments [39,41]. The data from different neutron diffraction studies reported in the literature performed on lipid mixtures of compositions closer to those used in our model were used for accurate comparison [36,38]. Schmitt et al. used a mixture of ceramide NP, ceramide AP, cholesterol and lignoceric acid at a molar ratio of 0.66:0.34:0.7:1, with ceramides consisting of C24 fatty acid tails [36]. Groen et al. performed an experiment with a mixture of different types of ceramides and free fatty acids and cholesterol, with most of the ceramides having C24 fatty acid tails [38]. The periodicities reported by Schmitt et al. [36] and Groen et al. [38] were 5.45 nm and 5.36 nm, respectively, which is close to our calculated value of 5.31 nm. To compare the molecular organization with experiments, the neutron scattering length density (NSLD) profiles generated from the simulation trajectories were compared with the reported profiles from neutron diffraction studies of Schmitt et al. [36] and Groen et al. [38]. Another model built using the same composition but with all ceramides in the hairpin conformation (Figure 6) was also adopted for comparative evaluation against experiment data. The hairpin-ceramide model is included only in this section for comparison. Altogether, the NSLD profiles of two in silico models—one built using ceramides in extended conformation and another built with ceramides in hairpin conformation—were compared with the experimental data from Schmitt et al. [36] and Groen et al. [38] (Figure 7).

The NSLD profiles suggest the orientation of the lipids arranged in lamellae [36,38]. High-density regions indicate the lipid polar groups, and low-density regions are due to the presence of -CH_2_ hydrocarbon tails [38]. The NSLD profile of the simple mixture of Schmitt et al. [36] was close to that of the near-native lipid mixture of Groen et al. [38]. This indicates that a simple model composed of ceramide NP, ceramide AP, lignoceric acid and cholesterol, as in Schmitt et al. [36], could represent a near-native organization. The hairpin-ceramide model forms two peaks at the polar region (near *z* = 0), whereas the extended-ceramide model forms a single peak, similar to the experiments. This is because in the hairpin-ceramide model containing 1.9 water/lipid, the lipid polar groups are separated by a layer of water, causing the lipids in different layers to form two distinct peaks. This comparison indicates that, for the estimated water content of 1.9 water/lipid, the head group arrangement in the extended-ceramide model is closer to that of the lipid phase organization observed in Groen et al. [38], who reported the same water content. The inconsistency between the experiment and the simulation in regions near z = ±1, where the cholesterol head groups are localized, has been reported in a previous study [42]. Furthermore, Schmitt et al. [36] used a molar ratio of 1:0.7:1 for ceramides, cholesterol and lignoceric acid, whereas our model employed a ratio of 1:1:1. Additionally, experiments by Groen et al. [38] and Schmitt et al. [36] observed phase-separated crystalline cholesterol, suggesting a lower incorporation of cholesterol into the studied ~5.3 nm phase. Future studies could explore reduced cholesterol concentrations to improve the model’s agreement with experimental observations.

**Figure 8:**
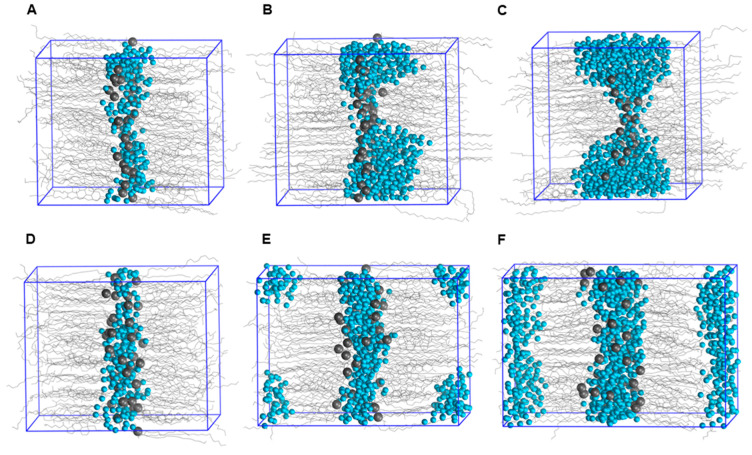
Localization of inserted water molecules in the extended-ceramide model: (**A**) No additional water, (**B**) 10 wt% water and (**C**) 20 wt% water. Localization of inserted water molecules in the hairpin-ceramide model: (**D**) No additional water, (**E**) 10 wt% water and (**F**) 20 wt% water. Lipid molecules are shown as gray lines, and water molecules are shown as cyan spheres. The nitrogen atoms in the polar head groups of ceramides are shown as gray spheres. These images are rendered from the last frames of production simulations.

To investigate the sensitivity of periodicity to hydration, water molecules were added to the extended-ceramide model and the hairpin-ceramide model at random locations, and periodicity was measured after equilibration. The equilibration and production simulations were performed using the protocol as described in Section 4.5. The periodicity of the hairpin-ceramide model increased substantially upon hydration from 5.40 nm to 6.00 nm with addition of 10 wt% water, and it increased to 6.62 nm with addition of 20 wt% water. This could be interpreted as swelling upon hydration. In contrast, the periodicity of the extended-ceramide model did not increase; rather, a slight decrease was observed from 5.31 nm to 5.27 nm and 5.06 nm upon addition of 10 wt% and 20 wt% water, respectively. This could be due to a slight increase in simulation box size in the *X* and *Y* dimensions. All water molecules were in the lipid head group region in the extended-ceramide model (Figure 8B,C). The localization of different components upon hydration can be identified using the density plot (Supporting Information: Figure S5). In the hairpin-ceramide model, water molecules were located in the lipid head group region, as well as in the hydrocarbon region (Figure 8E,F and Supporting Information: Figure S5B). After hydration, the density peaks of ceramide head groups in the hairpin-ceramide model drifted away from each other, showing an increase in interlayer spacing (Supporting Information: Figure S5B). The simulation results using the hairpin-ceramide model do not support the insensitivity of the stratum corneum lipid lamellae structure to hydration, as observed in experiments [33,34,35,36,37]. The extended-ceramide model better supports the observed hydration behavior of the stratum corneum lipid matrix. Better agreement of the extended-ceramide model with the compared experiments than the hairpin-ceramide model supports its utility for permeation studies.

### 2.3. Permeation Studies of Small Molecules Using the Model

Permeation studies were performed for three small molecules (water, benzene and estradiol) as test compounds because they represented a broad range of physicochemical properties. Water is hydrophilic; benzene is hydrophobic; and estradiol is a drug-like amphipathic molecule. The use of these three compounds helped us test how the model captures diverse permeation behaviors. 

**Figure 9:**
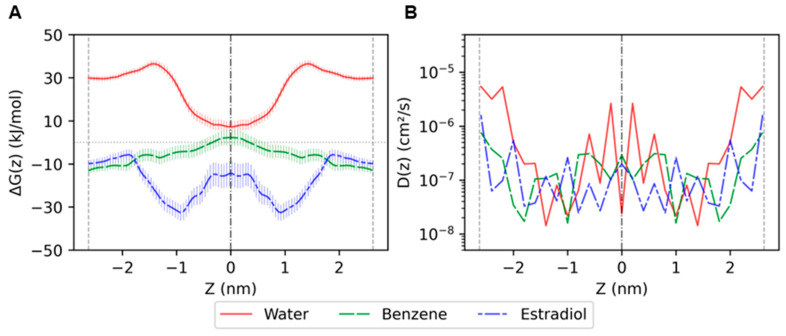
(**A**) Potential of mean force profiles and (**B**) diffusion coefficients of water, benzene and estradiol through the built model. Black dash-dotted lines indicate the center of the polar groups, and gray dashed lines indicate the hydrophobic core in the model. The error bars in (**A**) indicate standard deviation.

The permeability of the molecules through the stratum corneum is dependent on its solubility and diffusivity in the stratum corneum lipids. Solubility can be described using the free energy defined along the *Z* direction as the potential of mean force (PMF). The umbrella sampling method was used to generate the PMF and diffusion coefficients of the molecules. The PMF profiles of the studied molecules are shown in Figure 9A. The PMF landscape of the molecules through the bilayer is calculated relative to the free energy of the molecules in water, as described in Section 4.4. Δ*G_max_* is the highest value of the PMF and indicates the energy barrier associated with permeation. Water has Δ*G_max_* in the hydrocarbon tail region and the lowest Δ*G* in the lipid polar group region, as expected. For benzene, Δ*G_max_* is located in the lipid polar group region, and Δ*G* is low in the hydrocarbon tail region, as it is a hydrocarbon. Estradiol can be considered as a classic drug-like molecule with both hydrophobic and hydrophilic moieties. It has Δ*G_max_* in the hydrocarbon tail region and the lowest Δ*G* at *z* = ±1. The Δ*G* values of estradiol are all below zero in the system, indicating easier permeation. The Δ*G_max_* of benzene and estradiol is lower than that of water, as both are hydrophobic molecules. The diffusion coefficients of the molecules are in the range of 10^−8^–10^−5^ cm^2^/s (Figure 9B).

From the PMF and diffusion coefficients, the permeability coefficients were calculated using the inhomogeneous solubility–diffusion model (Equation (1)). Water had the lowest permeability coefficient, and benzene and estradiol had higher values than water due to their lipid solubilities (Table 1). Estradiol is an amphipathic molecule and had the highest permeability coefficient. The calculated permeability coefficient (log P) of water was in the range reported in diffusion cell experiments. However, the values of benzene and estradiol were higher compared to the experimental values. Higher permeability coefficients of hydrophobic molecules were reported in a study by Gupta et al. [29] as well. For benzene, the value predicted by Lundborg et al. [40] was closer to the experiments. The permeability coefficients calculated from our model increased with an increase in the octanol-water partition coefficients of the molecules (Table 1). This agrees with the Meyer–Overton rule [43], which states that the permeability coefficient is directly proportional to the partition coefficient from the aqueous into the organic phase. Although the permeability coefficients reported in the diffusion cell experiments could not be absolutely reproduced using MD simulations, the calculated PMF and permeability coefficients can be used for comparative evaluation of the intrinsic permeability of the molecules through the stratum corneum lipid matrix.

### 2.4. Mechanism of Action of Permeation Enhancers

**Figure 10:**
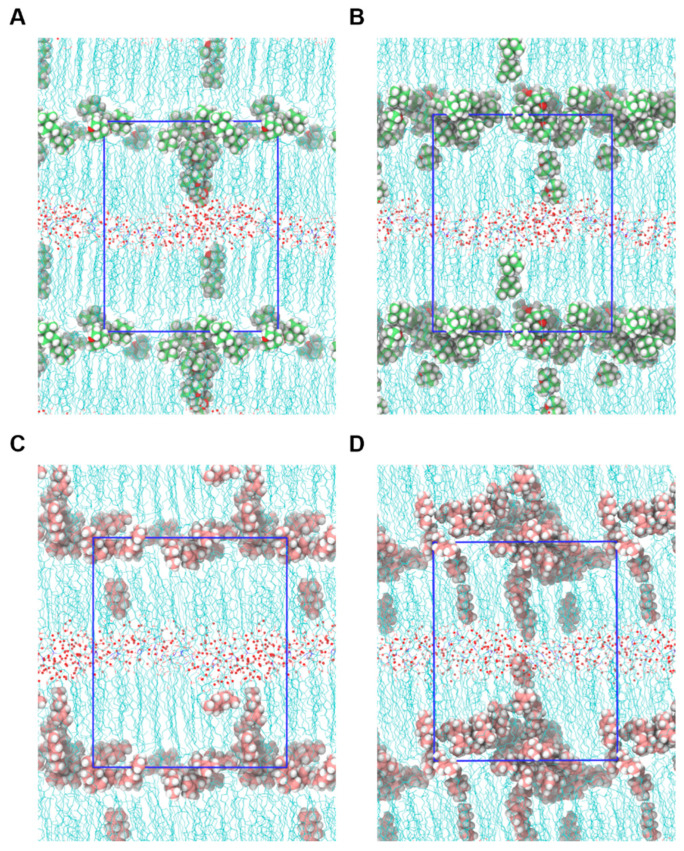
Snapshots from simulation trajectories showing the localization of permeation enhancers. Localization of eucalyptol in systems with concentrations of (**A**) 5 wt% and (**B**) 10 wt%. Localization of limonene in systems with concentrations of (**C**) 5 wt% and (**D**) 10 wt%. The oxygen atoms of lipids and water are shown in red; the carbon atoms of lipids, eucalyptol and limonene are shown in cyan, lime and pink, respectively; the hydrogen atoms of eucalyptol and limonene are shown in white; and the hydrogen atoms of water and lipids are not shown.

The mechanism of action of permeation enhancers—eucalyptol and limonene—was studied using MD simulations. Both of these molecules have been reported for their permeation-enhancing properties [63]. The permeation enhancers were added to the system with concentrations of 5 wt% and 10 wt%, approximately the concentrations used for skin permeation enhancers [63,64,65], and equilibration simulations were performed. Figure 10 shows the localization of eucalyptol and limonene in the system. Both molecules preferentially clustered in the lamellar interface region of hydrophobic tails through the simulation, as shown in the density plots (Figure S6B,D). With the increase in concentration of eucalyptol and limonene, a reduction in lipid tail density at the hydrocarbon interface can be observed (Figure S6A,C). This indicates that both molecules can disrupt the hydrophobic packing of the lipid matrix.

**Figure 11:**
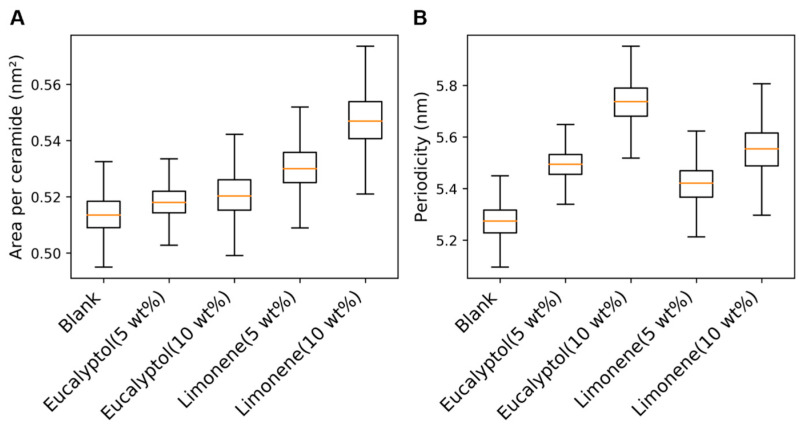
Properties of the stratum corneum lipid matrix systems equilibrated with permeation enhancers. (**A**) Area per ceramide values of the systems equilibrated with eucalyptol and limonene at concentrations of 5 wt % and 10 wt%. (**B**) Periodicity values of the systems equilibrated with eucalyptol and limonene at concentrations of 5 wt % and 10 wt%. ‘Blank’ refers to the system without permeation enhancers.

An increase in limonene concentration causes a notable increase in the area per ceramide values as compared to those of eucalyptol (Figure 11A). This is because more limonene molecules localize in the region between the lipid polar groups and the hydrocarbon interface (*z* = −2 to −1; *z* = 1 to 2) as compared to eucalyptol (Figure S6). An increase in area per ceramide is associated with increased disorder in lipid packing, which in turn enhances the diffusion of permeants. It can be interpreted that limonene being more lipophilic than eucalyptol causes a higher degree of disruption of the hydrophobic lipid packing in the stratum corneum. This agrees with the experimental findings that have suggested that the ability to disrupt hydrophobic packing increases with the rise in lipophilicity for terpene enhancers like eucalyptol and limonene [66].

An increase in periodicity was observed in the cases of both enhancers due to their localization in the hydrocarbon interface (Figure 11B). The lower increase in the periodicities of limonene-containing systems is due to the higher increase in their area per ceramide values as compared to eucalyptol-containing systems.

## 3. Discussion

Stratum corneum lipids are arranged in multiple layers to form alternating polar and hydrocarbon regions [12,15]. The developed model was built using the predominant lipid types in the stratum corneum to represent this motif. The model developed in this study is based on a view that the stratum corneum lipid matrix comprises alternating regions of lipid polar groups and hydrocarbon tails, with different periodicities arising as a function of lipid tail lengths. It was shown that the presented model agreed with neutron diffraction data and the hydration properties reported in the literature [36,38]. In silico atomistic models of the stratum corneum lipid matrix had been used previously in small molecule permeation studies [29,40]. The presented model uses a symmetric arrangement of ceramides as suggested by neutron diffraction experiments [36,38], unlike the model used by Lundborg et al. [40], which was based on cryo-electron microscopy data [16]. The permeability coefficients of three test compounds were calculated using the inhomogeneous solubility–diffusion model and compared with the permeability coefficients estimated from the diffusion cell experiments. Although the permeability coefficients reported in the diffusion cell experiments could not be reproduced for all the tested compounds, our simulations can be used to study the molecular mechanisms of permeation of any molecule, as our model represents a general structure of the stratum corneum lipid matrix validated by experimental data. In the current study, the focus was on validating the structure of the built model by testing the agreement of model properties like lipid organization and hydration behavior with the experimental data. In the future, it is proposed to study an expanded set of compounds with a wider range of molecular properties to understand the differences in the prediction of permeability coefficients with respect to the values obtained in diffusion cell experiments. Our model provides a mechanistic understanding of the action of permeation enhancers that can be tested using various experimental methods. This model can be replicated in its periodic images, and long simulations can be performed to understand the behavior of permeation enhancers in combination and their effect on the PMF of permeants. By coarse-graining the all-atom representation of the built model, the simulation time can be reduced to analyze a greater number of compounds.

## 4. Materials and Methods

### 4.1. Building the Model

The structures of all lipid molecules were generated using ‘3D Builder’ of Schrodinger Maestro 2021.3 [67]. The lipids and water molecules were packed using Packmol 18.169, which arranges molecules according to specified constraints [68]. The generated configuration contained 24 ceramide NP, 16 ceramide AP, 40 lignoceric acid, 40 cholesterol and 228 water molecules. All ceramide molecules were in the extended conformation and packed in the direction of the *Z*-axis with their polar groups in the middle of the box and their tails stretching to the periphery (Figure 1). Half the number of ceramide molecules were packed in the direction opposite to the other half with respect to the orientation of their sphingosine and fatty acid tails. Lignoceric acid and cholesterol molecules were packed along the *Z*-axis with their polar groups contacting ceramide polar groups and their tails extending to the periphery. The upper (*+z*) and lower (−*z*) halves of the box had equal numbers of ceramide sphingosine tails, ceramide fatty acid tails, lignoceric acid and cholesterol molecules. All water molecules were packed in the middle of the box contacting the polar groups of lipid molecules. All molecules were randomly distributed along the two other perpendicular axes. To ensure the robustness of our observations, three independent replicate systems were generated. These systems were identical in their lipid compositions and arrangements along the *Z*-axis but varied in the random distribution of lipids along the *X*- and *Y*-axes. This variability arises from the stochastic nature of Packmol’s packing algorithm [68], which randomly positions molecules within the defined constraints.

### 4.2. Equilibration of the Built Model

All simulations in this study were performed in GROMACS 2020.4 using CHARMM36 forcefield [69,70,71]. The CHARMM TIP3P model was used for water molecules. The particle mesh Ewald method with a cutoff of 1.2 nm was used to calculate electrostatic interactions [72]. Van der Waals interactions were truncated at a cutoff distance of 1.2 nm with a switching function applied at 1 nm. The system was periodic in all directions of the rectangular water box.

Energy minimization was performed over a total of 30,000 steps using the steepest descent algorithm in three distinct stages of 10,000 steps each, gradually relaxing the specific restraints applied to the system. Initially, position restraints were imposed on the heavy atoms of lipids and water molecules, with a force constant of 1000 kJ/mol/nm^2^. In the second stage, the same restraints were applied but only on the heavy atoms of lipid head groups and water molecules. In the final stage, all restraints were removed, allowing the system to relax completely. In each stage, the system was considered converged when the maximum force on any atom was reduced below 10.0 kJ/mol/nm or when the minimization reached the maximum limit of 10,000 steps. This energy minimization was followed by an equilibration phase in the canonical ensemble, maintaining a constant number of particles, volume and temperature (NVT). A time step of 1 fs was used for NVT equilibration. The temperature was set to 303.15 K using a velocity-rescale thermostat with a time constant of 1 ps. NVT equilibration was performed in four steps. Each step was run for 20 ps with different position restraints. The force constant used for position restraints applied on all heavy atoms in the *Z* direction was 1000 kJ/mol/nm^2^ during all four steps of NVT equilibration. The force constants used for position restraints on all heavy atoms in the *X* and *Y* directions were 1000 kJ/mol/nm^2^, 500 kJ/mol/nm^2^ and 100 kJ/mol/nm^2^ during the first three steps. No position restraints were applied in the *X* and *Y* directions during the fourth step of NVT equilibration. After NVT equilibration, equilibration was performed using the isothermal–isobaric (NPT) ensemble over 8 steps for durations of 0.5 ns, 0.5 ns, 9 ns, 10 ns, 10 ns, 20 ns, 25 ns and 500 ns, serially. During NPT equilibration, the temperature was maintained at 303.15 K using a velocity-rescale thermostat with a time constant of 1 ps, and the pressure was set to 1 bar using a Berendsen barostat with a time constant of 5 ps. A time step of 1 fs was used in the first six steps of NPT equilibration and 2 fs in the seventh and eighth steps of NPT equilibration. The position restraints with force constants of 1000 kJ/mol/nm^2^, 500 kJ/mol/nm^2^, 100 kJ/mol/nm^2^, 50 kJ/mol/nm^2^, 25 kJ/mol/nm^2^, 10 kJ/mol/nm^2^ and 10 kJ/mol/nm^2^ were applied in all heavy atoms in the *Z* direction from the first to the seventh NPT step, respectively. During the eighth NPT step, no position restraints were applied. After NPT equilibration, a production run was performed in the NPT ensemble with a time step of 2 fs for 300 ns, during which system properties were assessed. During production, the temperature was maintained at 303.15 K using a Nosé–Hoover thermostat with a time constant of 1 ps, and the pressure was maintained at 1 bar using a semi-isotropic Parrinello–Rahman barostat with a time constant of 5 ps. The positions and velocities were saved every 10 ps during the production run.

The hairpin-ceramide model discussed in Section 2.2 was built using CHARMM-GUI Membrane Builder and equilibrated using the protocol recommended by the same server [73]. After generating the model, the energy minimization was 1000 steps using the steepest descent algorithm, followed by NVT equilibration and NPT equilibration. NVT equilibration was performed in two steps for 125 ps each, with a time step of 1 fs. The position restraints were applied on the nitrogen atom of the ceramides, the oxygen atom of the cholesterol and the hydroxyl oxygen of the lignoceric acid in the *Z* direction with force constants of 1000 kJ/mol/nm^2^ and 400 kJ/mol/nm^2^ during the first and second steps of NVT equilibration, respectively. Dihedral restraints were applied on lipids with force constants of 1000 kJ/mol/nm^2^ and 400 kJ/mol/nm^2^ during the first and second steps of NVT equilibration, respectively. During NVT equilibration, the temperature was maintained at 303.15 K using a Berendsen thermostat, with a time constant of 1 ps. After NVT equilibration, NPT equilibration was performed by adding a Berendsen barostat with a time constant of 5 ps to maintain the pressure at 1 bar. NPT equilibration was performed over four steps for 125 ps, 500 ps, 500 ps and 200 ns, respectively. During the first step of NPT equilibration, a time step of 1 fs was used, and for the later steps, 2 fs was used. The position restraints were applied on the nitrogen atom of the ceramides, the oxygen atom of the cholesterol and the hydroxyl oxygen of the lignoceric acid in the *Z* direction with force constants of 400 kJ/mol/nm^2^, 200 kJ/mol/nm^2^ and 40 kJ/mol/nm^2^ during the first three steps of NPT equilibration, respectively. Dihedral restraints were applied on lipids with force constants of 200 kJ/mol/nm^2^, 200 kJ/mol/nm^2^ and 100 kJ/mol/nm^2^ during the first three steps of NPT equilibration, respectively. No restraints were applied during the last stage of NPT equilibration. The simulation conditions for the production run were the same as those used for the extended-ceramide model.

### 4.3. Calculation of System Properties

The area per ceramide is calculated by multiplying the lengths of the box in the *X* and *Y* directions and dividing the product by the number of ceramides in the box. The ‘*θ*’ angles between the sphingosine tails and fatty acid tails of the ceramides were calculated from the positions of the nitrogen atom and the 18th carbon atoms of the sphingosine tails and fatty acid tails of each ceramide molecule using MDAnalysis toolkit version 2.7.0 [74]. The density plots of the model were calculated using the density function of GROMACS. Using the number density profiles of carbon, hydrogen, oxygen and nitrogen atoms along the *Z*-axis as input, the NSLD profiles were generated using SIMtoEXP software for comparison with experimental data [75]. The scattering length density values were normalized, and the profiles were recentered to position the center of the polar region at *z* = 0. The profiles of non-deuterated samples from the reported experiments were used [36,38].

### 4.4. Permeability Assessment of Small Molecules Using the Built Model

MD simulations using the umbrella sampling method were used to generate the PMF profiles and diffusion coefficients of the compounds across the lipid matrix [76]. Inhomogeneous solubility–diffusion model, as defined using the following relation, was used to calculate the permeability coefficients (*P*) from the PMF and diffusion coefficients [77,78].

(1) $$\frac{1}{P}=R_{eff}=\int_{z_{1}}^{z_{2}}\frac{e^{\beta ∆G(z)}}{D(z)}$$

Here, *R_eff_* is the effective resistivity; *β* is the inverse of the product of absolute temperature and Boltzmann constant; *z* is a collective variable describing the relative position of the permeant along the *Z*-axis; and ∆*G*(*z*) and *D*(*z*) are the PMF and diffusion coefficients of the solute as a function of *z*. See below to see how *D*(*z*) is derived in Equation (2).

Steered MD simulations were performed to generate the initial configurations of the molecule through the stratum corneum lipid matrix. The small molecules were inserted into the system at the edges of the simulation box, and they were pulled toward the polar groups in the center of the box. The ‘insert-molecules’ module of GROMACS was used to introduce the small molecules into the system [70]. After insertion, energy minimization was performed over 5000 steps, followed by short equilibration in the NVT ensemble for 50 ps and in the NPT ensemble for 100 ps. During this equilibration, the position restraints with a force constant of 1000 kJ/mol/nm^2^ were applied on the small molecule. After the short equilibration, the small molecule was pulled along the *Z* direction using an umbrella potential with a force constant of 500 kJ/mol/nm^2^ and a pulling rate of 0.00025 nm/ps. Configurations were extracted from the trajectory at an interval of 0.2 nm along the reaction coordinate ‘*z*’. A total of 16 windows were taken for umbrella sampling. Umbrella sampling simulations were performed in the NPT ensemble using an umbrella potential with a force constant of 500 kJmol^−1^nm^−2^ applied on the small molecule. The other simulation parameters were the same as those described for the production run in Section 2.2. The positions and forces along the reaction coordinate were saved every 10 fs. For each window, simulations were performed for 45 ns, and data from the last 25 ns were used for analysis. The umbrella sampling covered only one half of the simulation box along the *Z*-axis. As the system is symmetric in composition along the *Z*-axis, the values of PMF and diffusion coefficients calculated for one half were duplicated for the other.

The PMF was calculated using the weighted histogram analysis method (WHAM) [79,80]. Using WHAM, unbiased PMF can be constructed from biased umbrella sampling simulations. It was ensured that there was sufficient overlap in permeant distributions between the adjacent windows to overcome statistical errors. The following method developed by Hummer allows the calculation of diffusion coefficients of a harmonically restrained molecule from the timeseries of its positions along the reaction coordinate [81].

(2) $$D\left(z\right)=\frac{{var(z)}^{2}}{\int_{0}^{\infty }C_{zz}\left(t\right)dt}\;\;$$

In this relation, *var*(*z*) is the variance of *z*, and *C_zz_* is its autocorrelation function. Once the values of the PMF and diffusion coefficients are calculated, *P* is derived via direct substitution of these values in Equation (1).

To accurately calculate the permeability coefficient from water to the stratum corneum, the PMF must be computed relative to that of the permeant in bulk water. However, the system does not contain bulk water. For this purpose, the free energy associated with transfer of the permeant from bulk water to the hydrocarbon interface ‘∆*G_transfer_*’ was added to all values of the PMF profile. ∆*G_transfer_* was calculated using the following equation.

(3) $$∆G_{tranfer}={∆G}_{lipid}-{∆G}_{water}\;\;$$

Here, ∆*G_lipid_* is the binding free energy of the permeant to the hydrocarbon interface of the lipids, and ∆*G_water_* is the solvation free energy. ∆*G_lipid_* and ∆*G_water_* were calculated from free energy perturbation simulations through implementation of the Bennett acceptance ratio (BAR) in GROMACS [70,82]. To calculate the free energy change, the non-bonded interactions of permeants with the system were gradually turned on using a ‘lambda’ parameter that controls the degree of coupling. The number of ‘lambda’ steps was increased until the estimated statistical error became less than 1 kJ/mol. Finally, 21 lambda steps were used for all three studied compounds (Table S1). ∆*G_water_* was calculated by immersing the permeant in a rhombic dodecahedron water box of minimal size. ∆*G_lipid_* was calculated from the first umbrella sampling window, in which the permeant was located at the hydrocarbon interface of lipid tails. The statistical errors associated with PMF calculation were estimated using the bootstrapping technique implemented in GROMACS [80].

### 4.5. Studying the Mechanism of Action of Permeation Enhancers

Permeation enhancers were inserted into the system from the first frame of the production run at random locations using the ‘insert-molecules’ function in GROMACS [70]. Energy minimization was performed over 40,000 steps using the steepest descent algorithm, followed by equilibration in the NVT ensemble. NVT equilibration was performed using a time step of 1 fs for a duration of 120 ps in three stages, during which the position restraints applied on the heavy atoms were gradually released. During NVT equilibration, the temperature was set to 303.15 K using a velocity-rescale thermostat with a time constant of 1 ps. NPT equilibration was performed in three stages. The first two stages were performed for 2.5 ns and 5 ns, respectively. The applied position restraints were released through the first two stages of NPT equilibration, and no position restraints were used in the third stage. The third stage was performed for a minimum of 200 ns and until system properties like area per ceramide and periodicity converged. During NPT equilibration, the temperature was maintained at 303.15 K using a velocity-rescale thermostat with a time constant of 1 ps, and the pressure was set to 1 bar using a Berendsen barostat with a time constant of 5 ps. The simulation parameters for production were the same as those described for the production run in Section 4.2. The system properties were studied during production.

## 5. Conclusions

A molecular model was developed for the multilamellar arrangement of intercellular lipids in the stratum corneum. A permeability assessment of test compounds like water, benzene and estradiol was performed. The utility of the model for studying the mechanism of action of permeation enhancers was investigated. Due to the lack of experimental data that would define the spatial distribution of the studied permeation enhancers within the stratum corneum lipids matrix, the localization of the studied permeation enhancers as observed in the simulations could not be validated. However, the role of permeation enhancers eucalyptol and limonene in disrupting hydrophobic packing, as reported in the experiments, could be explained. This model could be used in future to study the permeability of drug molecules in the presence of permeation enhancers. Compared to the empirical models that can be used to predict skin permeability, atomistic simulations are advantageous, in that they can reveal unprecedented molecular details involved in the permeation of a molecule and provide hypotheses for experimental testing. Hence, in addition to the values of permeability coefficients, these simulations give insights for improving the skin permeability of a molecule. Such insights are significant for the active compounds that are at different stages of research for their clinical applications.

## Figures and Tables

**Table 1:** Permeability coefficients (*P*) of water, benzene and estradiol calculated using the built model compared with experimental values.

	Permeability Coefficients Calculated Using the Built Model *log P* (cm/s)	Range of Permeability Coefficients Reported in Experimental Studies *log P* (cm/s)	log K_OW_	Permeability Coefficients Reported in Previous MD Simulation Studies *log P* (cm/s)	
				Study 1 ′	Study 2 ″
Water	−6.45	−5.34–−7.08 ^a,b,c,d,e,f,g,h^	−0.5	−4.15	−8.26
Benzene	−0.11	−4.35–−4.51 ^i,j^	2.1	0.28	−4.56
Estradiol	1.16	−4.81–−6.68 ^c,k,l,m,n,o,p,j^	4	-	-

The octanol-water partition coefficients (*K_OW_*) were retrieved from the PubChem database [44,45]. ^a^ Astley et al., 1976 [46]; ^b^ Barber et al., 1992 [47]; ^c^ Mitragotri et al., 1996 [48]; ^d^ Bond et al., 1988 [49]; ^e^ Clowes et al., 1994 [50]; ^f^ Rigg et al., 1990 [51]; ^g^ Scott et al., 1991 [52]; ^h^ Barber et al., 1995 [53]; ^i^ Blank et al., 1985 [54]; ^j^ Mitragotri et al., 1995 [55]; ^k^ Galey et al., 1976 [56]; ^l^ Goodman et al., 1988 [57]; ^m^ Johnson et al., 1995 [58]; ^n^ Johnson et al., 1996 [59]; ^o^ Knutson et al., 1993 [60]; ^p^ Michaels et al., 1975 [61]. The experimental *log P* values were retrieved from the HuskinDB database [62]. ′ Gupta et al., 2016 [29]; ″ Lundborg et al., 2018 [40].

## Data Availability

The scripts and structures of lipid molecules used to build the model; the simulation setup files and CHARMM36 parameters for equilibrating the model using MD simulation; the structure of the model after equilibration; the python scripts, commands and input files for calculating system properties like periodicity, area per ceramide, density and NSLD plots; the simulation setup files for hydration simulations and permeation enhancer simulations; the simulation setup files and scripts used to perform steered MD simulations and umbrella sampling simulations; and the scripts used to calculate PMF, diffusion coefficients and permeability coefficients are available at https://github.com/TeamSundar/Stratum-Corneum-Model (accessed on 12 January 2025).

## Supplementary Materials

The following supporting information can be downloaded at: https://www.mdpi.com/article/10.3390/ijms26020674/s1.
