# Supplementary material for: Establishing a General Atomistic Model for the Stratum Corneum Lipid Matrix Based on Experimental Data for Skin Permeation Studies
Source: Int J Mol Sci. 2025 Jan 15;26(2):674. doi: 10.3390/ijms26020674 (PMC11765878; doi:10.3390/ijms26020674)
Supplement: Supplementary file 1 [file ijms-26-00674-s001.zip › ijms-3397901-supplementary.pdf]

Supporting Information

Establishing a General Atomistic Model for the Stratum Corneum Lipid Matrix Based on Experimental Data for Skin Permeation Studies

Navaneethan Radhakrishnan [1,2], Sunil C. Kaul [3], Renu Wadhwa [3], Lee-Wei Yang [2,4,5,6] and Durai Sundar [1,7,8,*]

1 Department of Biochemical Engineering and Biotechnology, Indian Institute of Technology (IIT) Delhi, New Delhi 110016, India
2 Institute of Bioinformatics and Structural Biology, National Tsing Hua University, Hsinchu 300044, Taiwan
3 AIST-INDIA DAILAB, National Institute of Advanced Industrial Science & Technology (AIST), Tsukuba, Ibaraki 3058565, Japan
4 Bioinformatics Program, Institute of Information Sciences, Academia Sinica, Taipei 115201, Taiwan
5 Physics Division, National Center for Theoretical Sciences, Taipei 106319, Taiwan
6 Biomedical Artificial Intelligence PhD Program, National Tsing Hua University, Hsinchu 300044, Taiwan
7 Yardi School of Artificial Intelligence, Indian Institute of Technology (IIT) Delhi, New Delhi 110016, India
8 Institute of Bioinformatics and Applied Biotechnology (IBAB), Bengaluru - 560100, India
* Correspondence: sundar@dbeb.iitd.ac.in

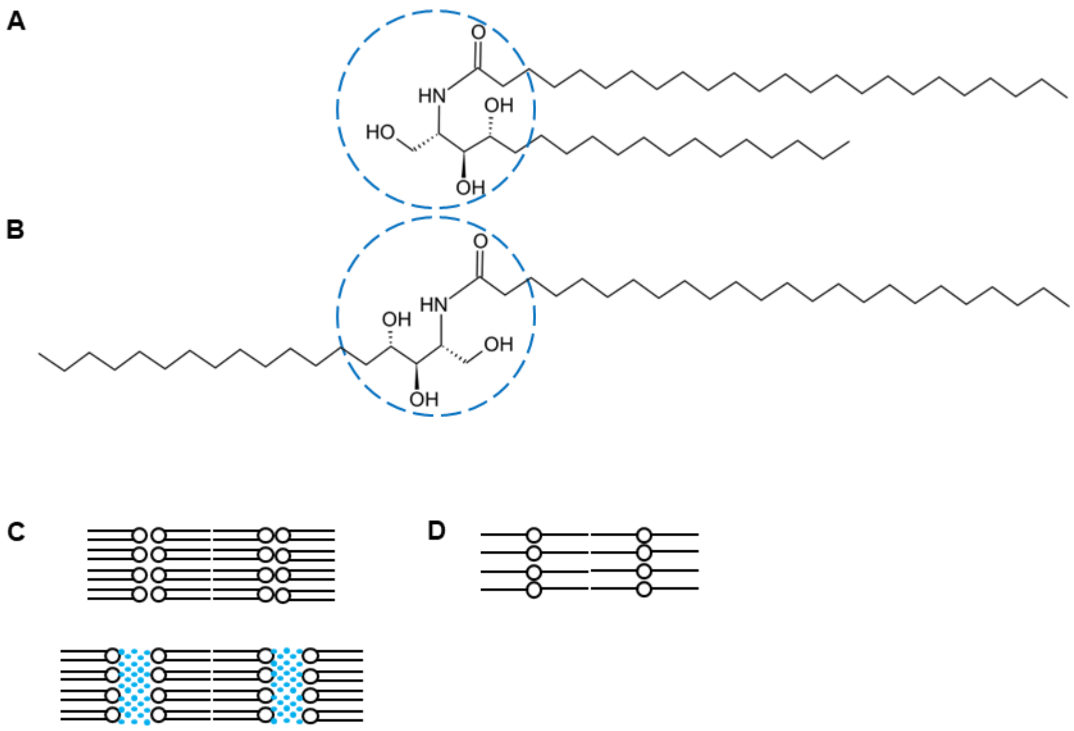

Figure S1. Two-dimensional representations of hairpin conformation (A) and extended conformation (B) of a ceramide molecule. Dashed blue circles show the polar head groups of the ceramide. (C) Schematic representation showing the swelling of two-tailed lipids in hairpin conformation. White circles denote the polar groups of lipids, and lines denote hydrophobic tails, blue dots denote the water. (D) Schematic representation showing the two-tailed lipids in extended conformation.

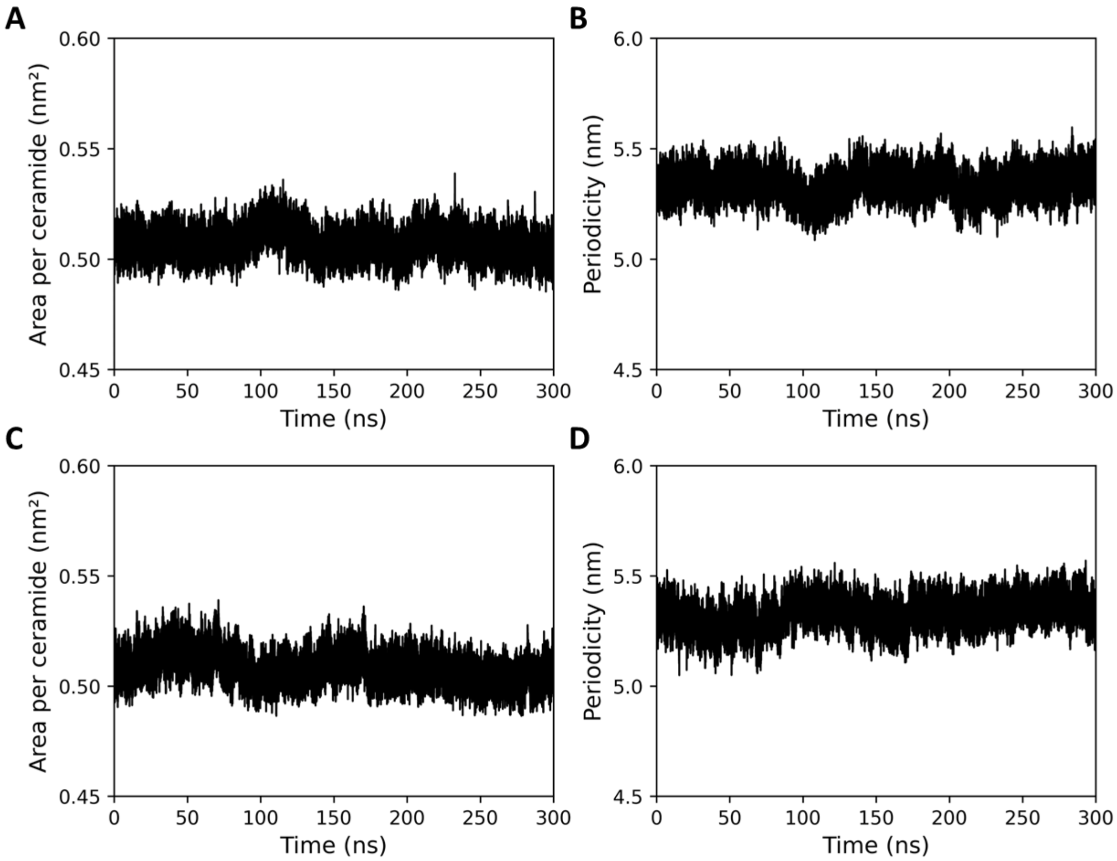

Figure S2. Properties of the two replicas of the built model of the stratum corneum lipid matrix during production simulation. Area per ceramide of replica 1 (A) and replica 2 (B); Periodicity of replica 1 (A) and replica 2 (B).

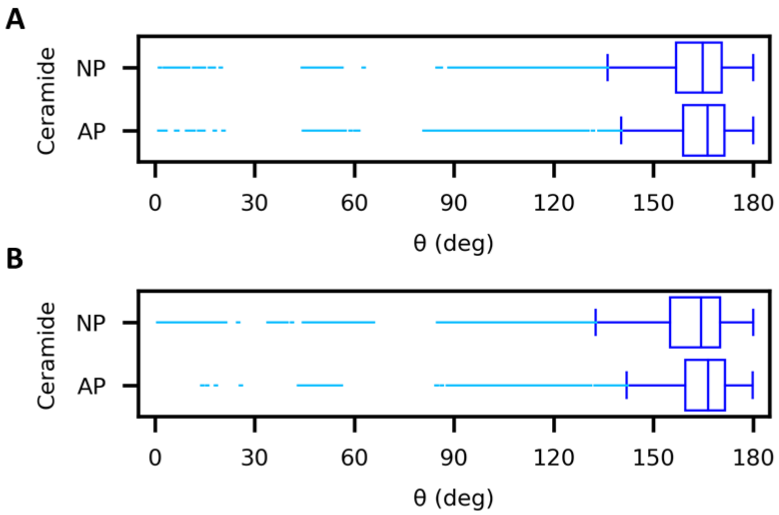

Figure S3. Angle distributions between the two tails of ceramide NP and ceramide AP molecules. θ indicates the angle between the sphingosine tails and fatty acid tails of ceramide molecules. The angle 'θ' between sphingosine tails and fatty acid tails of the ceramides were calculated from the positions of nitrogen atom and C18 atoms in the sphingosine tails and the fatty acid tails of each ceramide molecule.

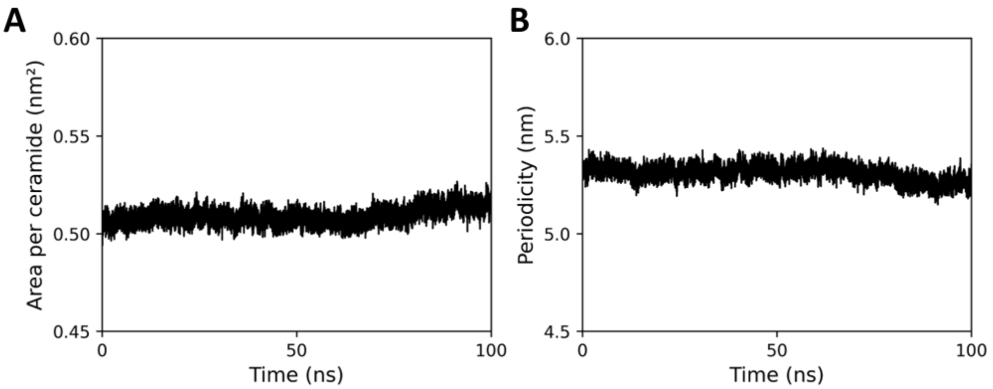

Figure S4. Properties of the system with 360 lipids during production simulation: Area per ceramide (A) and Periodicity (B).

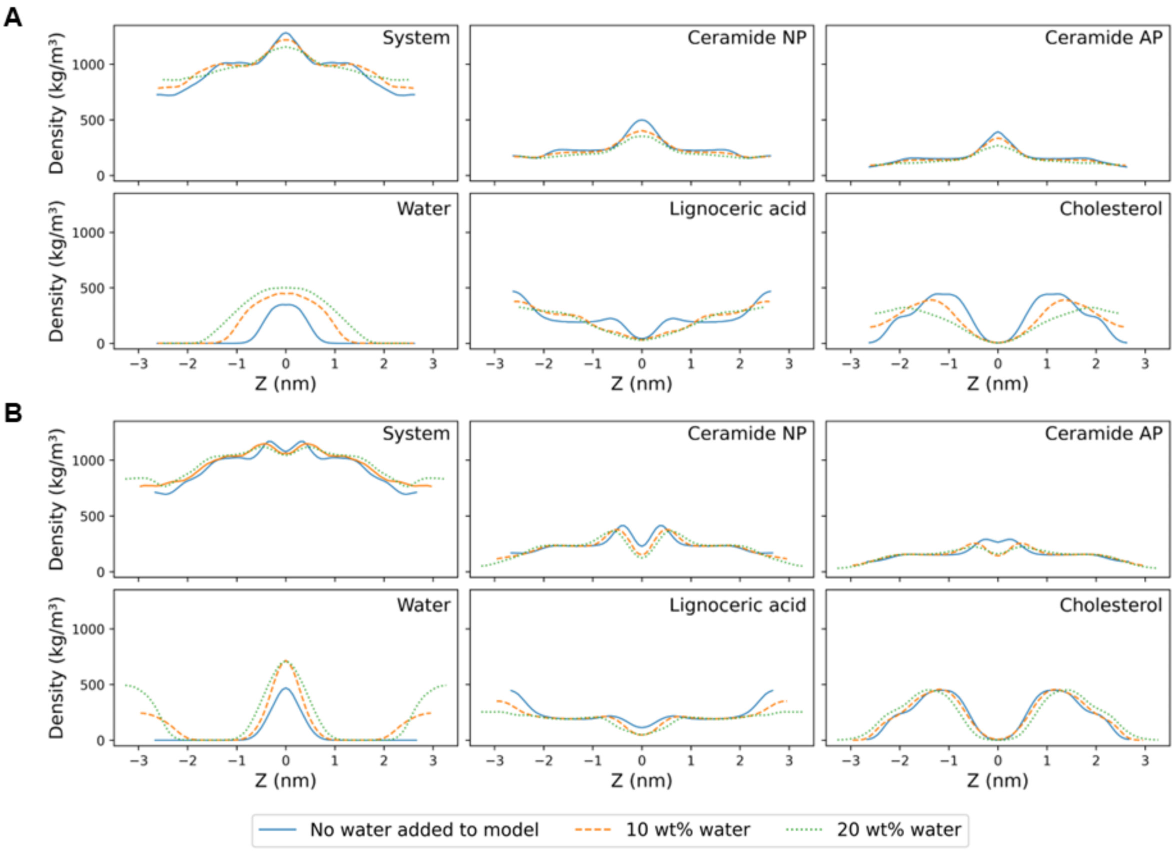

Figure S5. Densities of the system components of the model systems along the 'Z' axis upon hydration. (A) Ceramides in an extended conformation. (B) Ceramides in the hairpin conformation. 'No water' implies no additional water molecules have been added above the concentration of 1.9 waters/lipid.

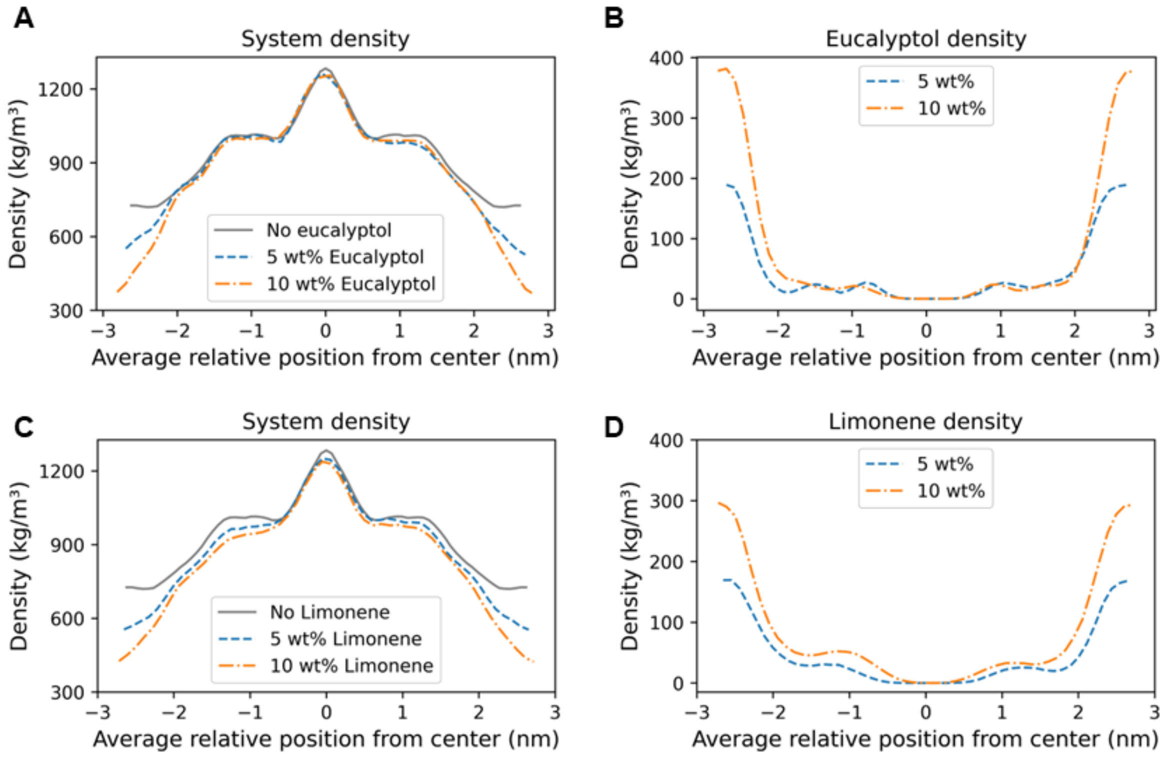

Figure S6. System density (A) and eucalyptol density (B) along the Z-axis after equilibration with eucalyptol. System density (C) and limonene density (D) along the Z-axis after equilibration with limonene. 'System density' indicates the density of system components (lipids and water) excluding the permeation enhancer.

Table S1: Values of the parameter 'lambda' used in the free energy perturbation simulations.

**Step**	**Lambda Value**
1	0
2	0.05
3	0.1
4	0.15
5	0.2
6	0.25
7	0.3
8	0.35
9	0.4
10	0.45
11	0.5
12	0.55
13	0.6
14	0.65
15	0.7
16	0.75
17	0.8
18	0.85
19	0.9
20	0.95
21	1
